# Case Report: Coinfection of *influenza A* and *Chlamydia psittaci* pneumonia

**DOI:** 10.3389/fmed.2026.1895006

**Published:** 2026-09-16

**Authors:** Binbin Feng, Ying Chen, Yueliang Chen, Jingjing Feng, Zhenwei Yu, Feng Guo, Jincheng Ruan

**Affiliations:** 1 Department of Critical Care Medicine, Sir Run Run Shaw Hospital, Zhejiang University School of Medicine, Hangzhou, Zhejiang, China; 2 Department of Pharmacy, Sir Run Run Shaw Hospital, Zhejiang University School of Medicine, Hangzhou, Zhejiang, China; 3 Department of Anus and Intestine Surgery, The TONGDE Hospital of Zhejiang Province, Hangzhou, Zhejiang, China

**Keywords:** *Chlamydia psittacosis* pneumonia, ECMO, *influenza A*, next-generation sequencing, omadacycline, severe pneumonia

## Abstract

The coinfection of influenza A and *Chlamydia psittaci pneumoniae* is rarely described in China. However, the clinical features of these diseases pose a significant diagnostic challenge. Therefore, it is necessary to report and review such a potentially fatal case. We report an 89-year-old male patient requiring admission to the intensive care unit with acute hypoxemic respiratory failure, shock and MODS. He presented with fever, fatigue, anorexia and dyspnea. The patient exhibited severe hypoxemia, shock, hepatic dysfunction (elevated liver enzymes and serum bilirubin), acute kidney injury and a SOFA score of 17. Mechanical ventilation (MV) and venous-venous extracorporeal membrane oxygenation (VV-ECMO) were established for respiratory support. The initial diagnosis focused on *influenza A* for his positive *influenza A* nucleic acid. However, the patient's imaging presentation was not coupled with viral pneumonia, which raised suspicion for other pathogens. Metagenomic next-generation sequencing (mNGS) detected coinfection of *C psittaci*, *influenza A H3N2* virus, and *Klebsiella pneumoniae* in bronchoalveolar lavage fluid. Concurrent targeted NGS (tNGS) detected *C. psittaci* in blood. Omadacycline and baloxavir marboxil were prescribed. The patient's condition improved, and he was successfully weaned from VV-ECMO and MV. He was discharged and recovered on day 60 of the follow-up. This case underscores the diagnostic complexity of coinfection and the use of NGS testing in this severe condition and the role of omadacycline in *psittacosis*.

## Introduction

*Chlamydia psittacosis* (CP) is a zoonotic pathogen that can cause severe pneumonia, respiratory failure, and acute organ dysfunction. This infection mainly results from contact with aerosols of bird or poultry urine, feces and excrement (1). The clinical manifestations vary from asymptomatic infection to severe atypical pneumonia. These nonspecific features often mimic viral and bacterial pneumonia, leading to diagnostic delays and inappropriate antimicrobial treatment during the influenza season. Meanwhile, the latest studies have shown that human-to-human transmission may occur (2, 3). The human-to-human situation is unclear, but close contact and infection severity may be risk factors according to the literature. The coinfection of *Chlamydia psittaci* and *influenza A* is extremely rare, and few cases have been reported. Here, we present a successfully cured case of severe pneumonia caused by *C. psittaci* and *influenza A* by VV ECMO with the use of next-generation sequencing (NGS).

## Case presentation

An 89-year-old male patient was transferred to our ICU from another hospital on December 23, 2025, with a 4-day history of high fever (39.5 °C), fatigue, anorexia and dyspnea. The patient had a medical history of hypertension and atrial fibrillation. He had no history of direct avian exposure. There were no chills, muscle soreness or limb weakness. Three days before admission, the patient was sent to the local hospital with low oxygen saturation. Then, he received a high-flow nasal cannula (HFNC). Meropenem (1 g every 8 h) and moxifloxacin were administered. Laboratory studies were abnormal, including WBC count (15.05 × 10^9^/L) with 96.1% neutrophils, high-sensitivity CRP (257.70 mg/L) and PCT (23.75 ng/mL). Biochemical tests revealed abnormal liver function: ALT 106.70 IU/L, AST 245.20 IU/L, and creatinine 112.00 µmol/L. Blood gas analysis indicated type I respiratory failure. Chest CT revealed multifocal inflammatory lesions in both lungs (prominent in the left lung). Tracheal intubation and mechanical ventilation were performed before transmission. Therefore, he was transferred to our intensive care unit (ICU) for further treatment. Norepinephrine (NE) was administered to maintain blood pressure.

Upon admission, the patient's physical examination was recorded as follows:

The temperature was 37.0 °C, blood pressure was 96/47 mmHg with continuing norepinephrine at 0.56 µg/kg/min, pulse rate was 99 breaths/min, pulse oxygen saturation was 93% with a fraction of inspired oxygen (FiO_2_) of 100%, and lung auscultation was wet rales on both lungs. There was no audible murmur on cardiac auscultation. Tenderness and hepatosplenomegaly were not detected. He had no neck stiffness. No rash was observed.

Laboratory data upon admission revealed a white blood cell (WBC) count of 13.2 ×  109/L with an elevated neutrophil ratio of 97.2% (Table 1). The concentration of C-reactive protein (CRP) and procalcitonin (PCT) were 198.6 mg/L and 59.04 ng/mL, respectively. Aspartate aminotransferase (AST) was 213 U/L, and alanine aminotransferase (ALT) was 99 U/L. The albumin level was 28 g/L. The creatinine level was 141 μmol/L. Arterial blood gas analysis showed a pH of 7.358, PO_2_ of 66.7 mmHg, PCO_2_ of 32.2 mmHg, and oxygenation index of 67 (Table 2). Chest imaging showed inflammation in both lungs, predominantly in the left lung, with partial consolidation of the left lung, accompanied by small bilateral pleural effusions (Figure 1 Day 1).

**Table 1:** Clinical laboratory results.

Measure	Reference range	D 1 (Dec 23)	D 2 (Dec 24)	D 3 (Dec 25)	D 4 (Dec 26)	D 5 (Dec 27)	D 6 (Dec 28)	D 7 (Dec 29)	D 9 (Dec 31)	D 11 (Jan 2)	D 13 (Jan 4)	D 15 (Jan 6)	D 17 (Jan 8)
WBC count (×10^9^/L)	3.5–9.5	13.2	15.5	13	12.2	12.9	12.4	9.3	7	9.2	10.4	5.2	6.2
Neutrophil count (×10^9^/L)	1.80–6.30	12.79	15.05	12.56	11.65	12.35	11.89	8.8	6.53	8.65	9.69	4.76	5.58
Neutrophil (%)	40.0–75.0	97.2	97.1	97	95.7	95.7	96.2	95.1	93.7	94	93.3	91.1	90.2
Hemoglobin (g/L)	130–175	123	120	90	83	81	80	75	72	85	92	86	87
Platelets (×10^9^/L)	125–350	90	81	68	53	47	38	37	52	72	76	85	106
D-dimer (ug/mL)	0–0.5	8.82	12.98	8.42	5.43	7.65	8.62	6.13	2.73	2.49	2.43	2.24	1.81
Na (mmol/L)	137–147	140	144	144	143	138	141	139	140	138	141	141	146
SCr(μmol/L)	57–111	141	124	96	80	83	82	67	55	102	83	70	67
K(mmol/L)	3.50–5.30	4.14	4.2	4.88	4.43	4.66	4.32	4.53	4.18	3.68	3.68	3.76	4.13
AST (U/L)	15–40	213	255	387	711	582	456	301	203	127	111	72	93
Albumin (g/L)	40–55	28	25.5	28.9	26.2	27.2	23.6	24.9	27.8	29	33.1	28.9	31.8
PCT (ng/mL)	<0.05	59.04	/	/	/	/	6.49	/	/	/	/	/	/
CRP (mg/L)	0–6	198.6	309.8	270	233.5	189.9	143.2	87.6	35.9	22.7	15.7	32.8	19

WBC, white blood cell; SCr, Serum Creatinine; AST, aspartate; PCT, procalcitonin; CRP, C-reactive protein.

**Table 2:** Ventilator and ECMO parameters.

	D 1 (Dec 23)	D 2 (Dec 24)	D 3 (Dec 25)	D 4 (Dec 26)	D 5 (Dec 27)	D 6 (Dec 28)	D 7 (Dec 29)	D 9 (Dec 31)	D 12 (Jan 2)	D 13 (Jan 4)	D 15 (Jan 6)	D 17 (Jan 8)
FiO_2_	100%	100%	100%	100%	100%	100%	100%	70%	50%	45%	40%	40%
PEEP (cmH_2_O)	12–14	12	12	12	14	14	12	12	10	10	8–6	/
Pplat	27	/	24	23	27	27	21	20	20	22	/	/
CL (mL/mmHg)	61	/	29	27	31	33	37	37	35	53	/	/
Vt (mL)	500-600	300-600	250-350	250-350	300-400	400-450	400-500	350-450	350-500	400-600	400-550	/
PCO_2_(mmHg)	32.2	38.1	45.6	40.2	43.7	36.2	25.4	36.1	39.5	35.6	37.9	30.1
PO_2_(mmHg)	66.7	71.6	122	91.5	115	158	180	152	155	127	153	101
P/F ratio (mmHg)	67	72	122	92	115	158	180	217	310	282	383	253
ECMOration rate(revolutions/min)	/	2,500	2,500	2,500	2,500	2,500	2,500	2,500	2,300	/	/	/
ECMO flux (3 L/min)	/	3.2	3.2	3.3	3.3	3.2	3.2	3.3	2.7	/	/	/
ECMO FiO_2_	/	100%	100%	100%	100%	100%	100%	60%-40%	40%-0	/	/	/

ECMO, extracorporeal membrane oxygenation; FiO_2_, fraction of inspired oxygen; PEEP, positive end-expiratory pressure; Vt, tidal volume; Pplat, platform pressure; CL, lung compliance; PCO_2_, partial pressure of carbon dioxide; PO_2_, partial pressure of oxygen. P/F ratio,PaO₂/FiO₂ ratio.

**Figure 1:**
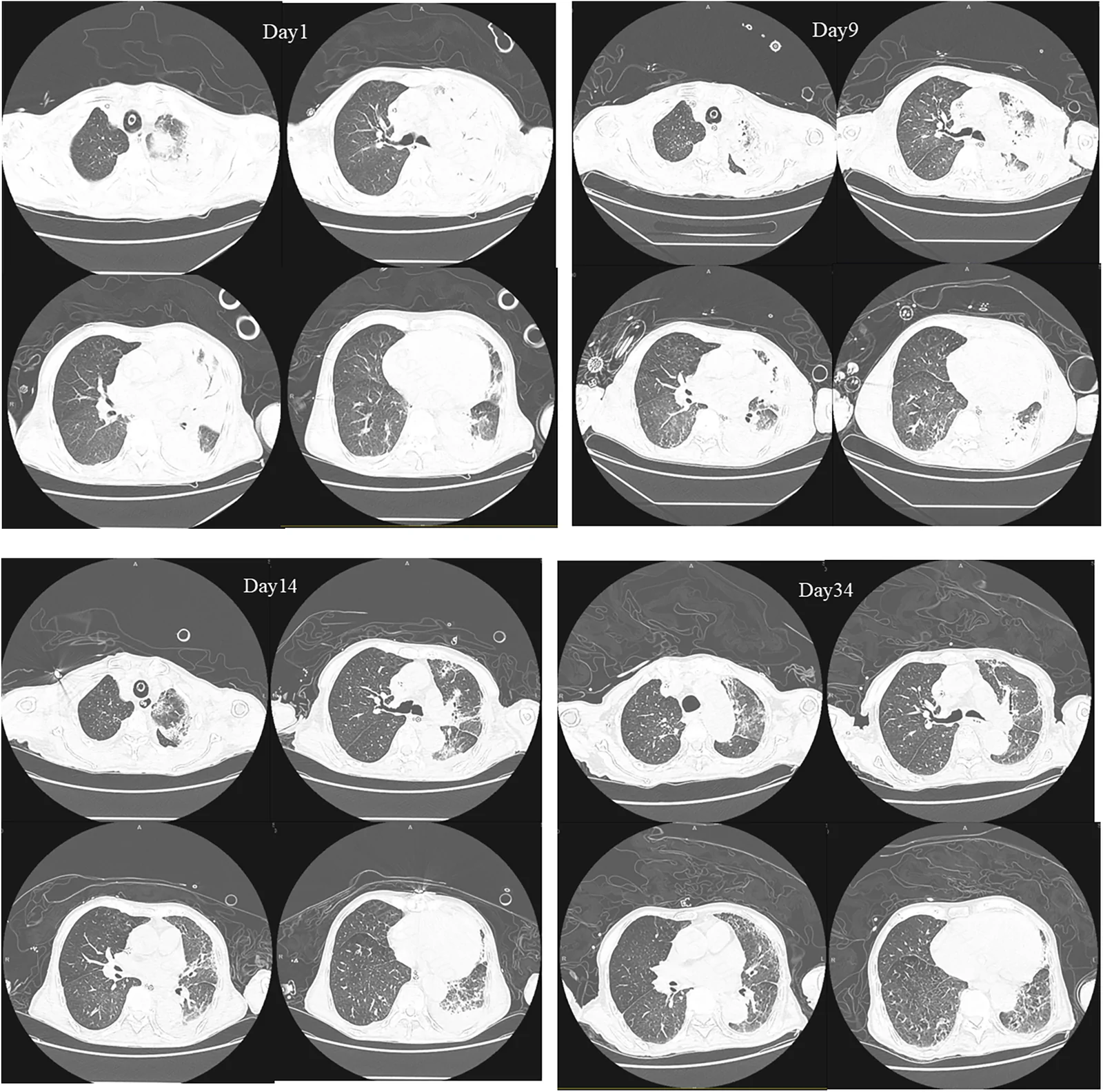
Serial chest CT imaging. (Day 1) Initial CT on Dec 23 demonstrating consolidation in both lungs, predominantly in the left lung, and small bilateral pleural effusions. (Day 9) CT on Dec 31 showing partial improvement in consolidation. (Day 14) CT on Jan 5 demonstrating significant interval resolution. (Day 34) CT on Jan 24 showing marked resolution.

The APACHE II and SOFA scores on admission were 16 and 17, respectively.

The patient was diagnosed with community-acquired pneumonia, and empirical intravenous antibiotic therapy was started with meropenem**,** omadacycline and caspofungin**.**

Prone positioning was initiated, but the patient's oxygenation did not improve. The patient received continuous renal replacement therapy (CRRT) for acute kidney injury. A Pulse index continuous cardiac output (PiCCO) catheter was placed for volumetric monitoring, and fluid balance was carefully adjusted based on the measurements. After these treatments, hemodynamics and oxygenation remained unchanged. The dosage of vasopressors was increased to NE 0.94 µg/kg/min plus epinephrine 0.07 µg/kg/min, and the repeated ABG demonstrated no improvement in oxygenation.

On day 2, refractory hypoxemia with PO_2_/FiO_2_ was 72, and none of the initial treatments could alleviate the patient's clinical condition. Therefore, VV ECMO was commenced. VV ECMO was quickly established through femoral artery-internal jugular vein catheterization to provide respiratory support. The rotational speed of ECMO was set at 2500 RPM with a flow rate of 3.0 L/min (Cardiohelp, MAQUET, Germany). Additionally, the prone position was performed after the establishment of VV ECMO. With the assistance of MV and VV ECMO (Table 2), the patient's oxygenation and circulation improved significantly, and a small dose of NE was required.

The patient was prescribed the antiviral medication baloxavir marboxil for positive *influenza A* nucleic acid testing. However, the patient's imaging presentation was not coupled with viral pneumonia, which raised suspicion for other pathogens. To clarify the pathogen, BALF was collected via flexible bronchoscopy on the 1st day of admission, and blood targeted NGS was performed. The mNGS and tNGS was performed by a professional, commercially available testing service (4). On the 3rd day of the ICU stay, the mNGS test revealed the presence of Chlamydia psittaci (Relative abundance: 77.08%), influenza A virus H3N2 subtype and Klebsiella pneumoniae. The blood tNGS results showed Chlamydia psittaci, and no other pathogenic bacteria, viruses, or fungi were detected (Table 3). The positive nucleic acid testing, BALF mNGS and blood tNGS confirming the etiological diagnosis. While concurrent sputum and blood cultures were negative in bacteria and fungi.

**Table 3:** Next-generation sequencing results.

	Sample	List of Detected Pathogens Reads and Relative abundance(%)
D3	BALF	*Chlamydia psittaci* 61070 77.08 *Influenza A virus H3N2* subtype 270 0.34 *Klebsiella pneumoniae* 14313 18.07
D4	Blood	*Chlamydia psittaci* 253
D7	BALF	*Chlamydia psittaci* 732 2.31 *Klebsiella pneumoniae* 10804 34.03 *Candida albicans* 925 2.91

BALF, bronchoalveolar lavage fluid.

Simultaneously, antibiotic therapy was adjusted to intravenous ceftazidime-avibactam and omadacycline (Figure 2). The patient's clinical condition gradually improved. Repeated BALF mNGS tests on the 7nd day showed *Chlamydia psittaci* (Relative abundance: 2.31%), *Klebsiella pneumoniae*, and *Candida albicans* (Table 3).

**Figure 2:**
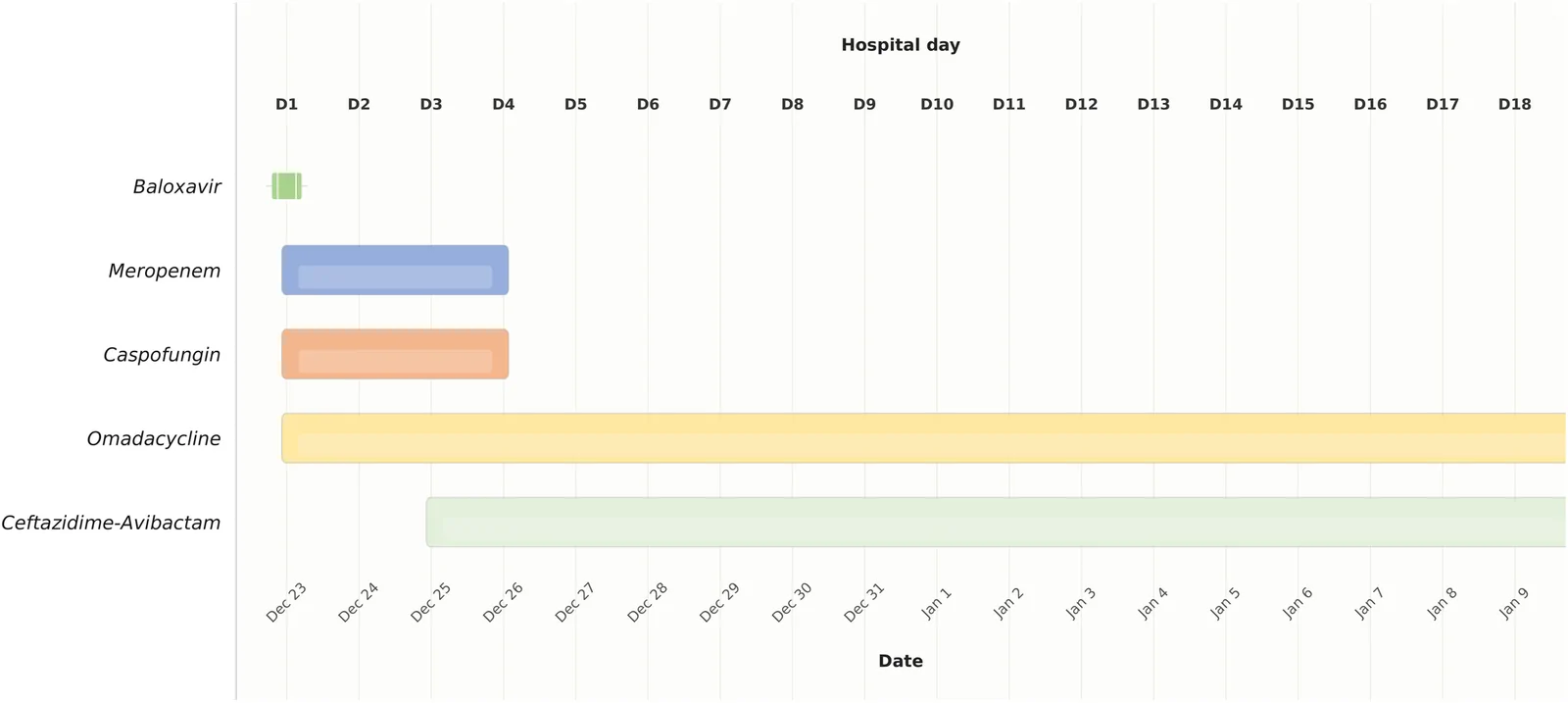
Antimicrobial treatment courses during hospitalization.

The patient's vasopressor dosage and ventilator and VA-ECMO settings gradually decreased. Furthermore, the patient's infection biomarkers gradually decreased (Table 1), and the lung CT scan demonstrated a gradual absorption of the infiltrative lesions (Figure 1).

On the 11th day of the ICU stay, VV-ECMO was successfully withdrawn. On the 15th day of the ICU stay, the patient's consciousness was clear, and he passed the spontaneous breathing test. MV and endotracheal tubes were successfully discontinued and removed, respectively. The patient's vital signs were stable, and the organ function had recovered. The patient was transferred to the ward for further rehabilitation. Omadacycline was stopped on the 17th day.

A telephone follow-up 60 days later confirmed sustained recovery without recurrence. Epidemiological investigation revealed that pet parrots were kept by residents in the same community, although the patient firmly denied any direct contact with these birds or their owners. No additional psittacosis cases were identified in the community.

## Discussion

In this case, we report an 89-year-old male patient requiring admission to the intensive care unit with acute respiratory failure, septic shock, acute kidney injury and MODS. Mechanical ventilation (MV) and venous-venous extracorporeal membrane oxygenation (VV-ECMO) were established for respiratory support. Although laboratory tests confirmed *influenza A* infection, from a clinical perspective, the severity of pneumonia suggested an alternative or additional pathogen. The mNGS test quickly identified *CP* gene fragment and *influenza A* gene fragment in BALF, and tNGS test identified *CP* gene fragment in blood, which strongly support the diagnosis of *coinfection of influenza A* and *Chlamydia psittaci*. Based on these results, targeted antimicrobial therapy (including omadacycline and baloxavir marboxil) was prescribed. After targeted antibiotics and organ support treatment, pneumonia and MODS gradually improved, and the patient was successfully weaned from VV-ECMO and MV. Finally, the elderly patient recovered well and was discharged.

This case highlights challenges in diagnosis of coinfection with psittacosis and *influenza A*: the absence of a typical exposure history, nonspecific initial manifestations during influenza season, and the limitations of conventional diagnostic techniques. The coinfection could not be distinguished from severe influenza pneumonia clinically. The symptoms of *psittacosis* and *influenza A* overlap substantially, making early differentiation impossible in clinical practice during influenza season. Moreover, conventional diagnostic tests were inadequate: the rapid antigen test for *influenza A* has limited sensitivity, and routine sputum culture failed to detect *C. psittaci*.

The coinfection of *Chlamydia psittaci* (*C. psittaci*) and *other* pathogens is unusual. Only cases have been reported (5, 6). One study found that more coinfecting pathogens were found in *C. psittaci* and had a higher risk of mixed infection, which in turn led to more severe clinical symptoms and a longer disease course cycle (7). One study found a higher coinfection rate of 91.7% (11/12) in the severe group (8). Several factors contributed to the delayed recognition of coinfection with *Chlamydia psittaci*.

Early identification of pathogens can significantly improve the prognosis. mNGS is advised to be applied to severe pneumonia patients as early as possible in addition to conventional methods to improve the prognosis and reduce the length of hospital stay (9). The use of mNGS can improve accuracy and reduce the delay in the diagnosis of *psittacosis*. Severe *psittacosis* pneumonia responds well to the timely use of appropriate antibiotics (10).

Treatment options for *psittacosis* include tetracyclines, macrolides, and fluoroquinolones. Omadacycline, a novel 9-aminomethylcycline derivative of minocycline, belongs to the tetracycline family and exhibits broad-spectrum antibacterial activity, including against Chlamydia (11, 12). However, currently, there is a lack of extensive clinical data and systematic evaluation regarding the use of omadacycline in *psittacosis* treatment. The value of omadacycline in the treatment of severe *psittacosis* pneumonia has been proven by multiple case reports. Chen and colleagues reported a case of an 86-year-old man with severe *psittacosis* treated with intravenous omadacycline (200 mg as a loading dose, followed by 100 mg daily). The patient's symptoms and chest CT improved significantly (13). In another case report, omadacycline was successfully prescribed for patients with severe *psittacosis* pneumonia complicated by septic shock (14). Together, these cases provide further support for the efficacy and safety of omadacycline in the management of *psittacosis*. Wang and colleagues observed sixteen patients with severe *psittacosis* pneumonia and described omadacycline, providing a better understanding of effectiveness in severe *psittacosis* pneumonia with ARDS (15). This highlights the necessity for further research on the role of omadacycline in *psittacosis* treatment (15).

## Conclusions

Severe coinfection of *CP* and *influenza A* is not common during the influenza season. Early diagnosis, targeted antibiotic therapy, and organ support contribute to facilitate disease recovery. This case provides a reminder that clinicians should suspect coinfection and emphasizes the importance of NGS and the role of omadacycline in *psittacosis*.

## Data Availability

The original contributions presented in the study are included in the article/Supplementary Material, further inquiries can be directed to the corresponding authors.
